# Inflammatory Effects of High and Moderate Intensity Exercise—A Systematic Review

**DOI:** 10.3389/fphys.2019.01550

**Published:** 2020-01-09

**Authors:** Érica Cerqueira, Daniel A. Marinho, Henrique P. Neiva, Olga Lourenço

**Affiliations:** ^1^FCS – UBI, Faculty of Health Sciences, University of Beira Interior, Covilha, Portugal; ^2^Department of Sport Sciences, University of Beira Interior, Covilha, Portugal; ^3^Research Center in Sport Sciences, Health Sciences and Human Development (CIDESD), Covilha, Portugal; ^4^CICS – UBI, Health Sciences Research Centre, University of Beira Interior, Covilha, Portugal

**Keywords:** inflammation, exercise, high intensity, moderate intensity, cytokines

## Abstract

**Background:** Exercise leads to a robust inflammatory response mainly characterized by the mobilization of leukocytes and an increase in circulating inflammatory mediators produced by immune cells and directly from the active muscle tissue. Both positive and negative effects on immune function and susceptibility to minor illness have been observed following different training protocols. While engaging in moderate activity may enhance immune function above sedentary levels, excessive amounts of prolonged, high-intensity exercise may impair immune function. Thus, the aim of the present review was to clarify the inflammatory effects in response to different exercise intensities.

**Methods:** Search was performed on PubMed and was completed on July 31st, 2017. The studies were eligible if they met the predefined inclusion criteria: a) observational or interventional studies, b) conducted in healthy adults (18–65 years), c) written in Portuguese, English or Spanish, d) including moderate and/or intense exercise. Eighteen articles were included. The specific components that were examined included circulating blood levels of cytokines, leukocytes, creatine kinase (CK) and C-reactive protein (CRP). The methodological quality of the included studies was assessed.

**Results:** Most of the intervention studies showed changes in the assessed biomarkers, although these changes were not consistent. White blood cells (WBC) had an increase immediately after intensive exercise (> 64% VO_2max_), without alteration after moderate exercise (46–64% VO_2max_). The results suggested an elevation of the pro-inflammatory cytokines, namely IL-6, followed by an elevation of IL-10 that were more evident after intense exercise bouts. CRP increased both after intense and moderate exercise, with peak increases up to 28 h. CK increased only after intensive and long exercising.

**Conclusion:** In summary, intense long exercise can lead, in general, to higher levels of inflammatory mediators, and thus might increase the risk of injury and chronic inflammation. In contrast, moderate exercise or vigorous exercise with appropriate resting periods can achieve maximum benefit.

## Introduction

Inflammation is characterized by a cascade of cellular and molecular events leading to an increase in body temperature, capillary dilatation, and production of blood-borne soluble components (Allen et al., 2015; Gonzalo-Calvo et al., 2015). These responses, which can be induced by stressors and are vital for host defense and natural tissue homeostasis, initiate the elimination of noxious compounds and damaged tissue (Moldoveanu et al., 2001; Allen et al., 2015).

Exercise works as a stressor during and after its execution and it is able to cause inflammation (Silveira et al., 2016). Interestingly, however, regular physical exercise training may be considered a long-lasting anti-inflammatory therapy, after the acute inflammatory actions are resolved (Gleeson et al., 2011; Allen et al., 2015). Moreover, pro-inflammatory processes that occur after exercise, such as increases in the expression of pro-inflammatory cytokines, may be vital for the long-term adaptive responses to exercise training. Inflammation is essential in order to repair processes to occur, like those resulting from exercise and training (Oishi and Manabe, 2018). Consequently, exercise-induced changes in inflammation can be divided into acute effects (changes during and immediately following a bout of exercise) and long-term effects (changes in resting or basal levels, when the acute exercise-induced effects have been washed out) (Roca-rodríguez et al., 2015; Pedersen, 2017).

Some authors have suggested that acute exercise bouts initiate a complex cascade of inflammatory events, which depend on the type, intensity, duration and familiarity of the exercise, as well as the age and clinical condition of the participants (Moldoveanu et al., 2001; Allen et al., 2015; Bigley and Simpson, 2015; Silveira et al., 2016; Hennigar et al., 2017; Peake et al., 2017). Measurable immune parameters affected by exercise comprise changes in peripheral blood cell numbers, granulocyte activity, NK cell cytotoxic activity, lymphocyte proliferation, and cytokine levels in plasma, among others (Moldoveanu et al., 2001; Petersen and Pedersen, 2005; Timmons and Cieslak, 2008; Gleeson et al., 2011; Allen et al., 2015; Bigley and Simpson, 2015; Lightfoot and Cooper, 2016; Hennigar et al., 2017; Peake et al., 2017; Shaw et al., 2017).

Cytokines are soluble proteins or glycoproteins, produced and segregated during inflammation, that mediate the communication between immune and non-immune cells and regulate biological processes (Chen et al., 2018). The production of cytokines can be upregulated rapidly in response to inflammatory stimuli, and this response can be transient or prolonged (Allen et al., 2015). The pro-inflammatory cytokines (TNF-α, IL-1β, and IL-6) are released after physical activity of sufficient intensity, followed by the release of anti-inflammatory or regulatory cytokines (IL-4, IL-10, IL-1RA, and IL-13) that attenuate that response (Moldoveanu et al., 2001).

Several proteins are affected in response to inflammatory processes, the majority showing increased levels shortly after an inflammatory reaction (Fedewa et al., 2016). Those proteins whose concentration increases are referred to as positive acute-phase proteins. CRP is a hepatic acute-phase protein, a marker of systemic inflammation and is associated with cardiovascular risk (Allen et al., 2015). Moreover, its levels have been correlated with frailty, morbidity, and mortality (Allen et al., 2015). CRP has lower levels in people who do moderate exercise compared to inactive people (Allen et al., 2015; Fedewa et al., 2016). Creatine kinase (CK) is a protein involved in muscle metabolism, and its concentration is generally considered a physical stress marker (Moghadam-Kia et al., 2016). Leakage of CK into the plasma is accepted as a semi-quantitative indicator of muscle fiber damage (Marqués-Jiménez et al., 2016). CK levels have a significant variation with sex and race and also with exercise type: eccentric exercise causing more muscle damage than concentric contractions of the same vigor (Baumert et al., 2016; Moghadam-Kia et al., 2016).

Overall, it seems evident that there are immune changes after exercise, especially with increased intensity. Moreover, there is a belief that these changes differ markedly after heavy exercitation from those following moderate exercise. Therefore, this systematic review aimed to synthesize and analyze the moderate and intense physical activity in healthy active adults, to explore the associated inflammation markers, and to provide quantitative estimates on the change of these markers.

## Methods

### Search Strategy

A comprehensive search in the MEDLINE (PubMed) database was conducted (NCBI)^1^ The main target was to find studies that described immunologic changes in response to moderate and/or intense/vigorous exercise. The search expression (“Inflammation”[Mesh]) AND (“Exercise”[Mesh]) was used in all fields (Table 1). No limitation was made in publication date or duration of the study. Literature published from the inception of the database up to 31 July 2017 was included.

**Table 1:** Search strategy and inclusion/exclusion criteria based on PICO.

**Databases**	**Search terms**	**PICO**	**Inclusion criteria**	**Exclusion criteria**
PubMed	Inflammation Exercise	Population	Healthy adults (18–65 years)	Sedentary adults; adults with disease
		Intervention	Moderate exercise	No exercise intensity definition
			Intense exercise	
		Comparison	Intense with moderate exercise	
		Outcome	Alterations in PBMC (WBC, lymphocytes, NK cells, or NK cytolytic activity), Cytokines (IL-6, IL-8, IL-1β, TNF-α, or IL-10), CRP, or CK	No results on inflammatory markers

### Eligibility Criteria

After the initial search, duplicates and studies not relevant for this analysis were excluded and the remaining studies' abstracts were examined by two independent reviewers. Doubts regarding the inclusion or exclusion of studies were resolved by discussion between the two independent researchers. After this first selection, both researchers read through the articles to decide whether they were eligible using criteria defined with the PICO (Population, Intervention, Comparison, and Outcome) criteria (Table 1; Methley et al., 2014). Further studies were considered for inclusion after verifying the references of the original studies. As no other databases were searched, manual searches were performed in the reference lists of all included studies and relevant review studies.

### Risk of Bias Assessment

Scientific quality of the studies was assessed independently by two reviewers using the STROBE scale for cross-sectional studies and the CONSORT scale for clinical trials (von Elm et al., 2008; Schulz and Atlman, 2010). If assessment outcomes were conflicting, a consensus-based final score was attributed.

### Data Extraction and Analysis

This systematic literature review about inflammatory effects following high and moderate intensity exercise was registered in PROSPERO (CRD 42018085835) and was performed according to the recommendations established by the Preferred Reporting Items for Systematic reviews and Meta-Analysis (PRISMA) statement guidelines (Urrútia and Bonfill, 2010).

The main participants' characteristics and the main study outcomes were identified. Data regarding type, intensity and duration of physical exercise and exercise-induced changes in inflammation markers were identified and appraised. Moderate exercise was defined by: Borg scale between 12 and 13 or % of maximal heart rate (HR_max_), between 64 and 76% or % maximal heart rate reserve (HR_max_), between 40 and 60% or % maximal oxygen consumption (VO_2max_), between 46 and 64% or metabolic equivalent (MET), between 3 and 6 or % repetition maximum (RM), between 50 and 70% or MET by age between 4.8 and 7.2 (young: 20–39 years) and 4.0–6.0 (middle age: 40–64 years). Intense exercise was defined by: Borg scale > 13 or HR_max_ > 76% or HR_max_ >60% or VO_2max_ > 64% or MET > 6.0 or RM > 70% or MET by age > 7.2 (young: 20–39 years) and > 6.0 (middle age: 40–64 years) (Pescatello et al., 2013). The effects of the exercise intensities on inflammatory markers were evaluated in blood samples collected before and after the exercise bouts and relative increases related to the baseline levels were determined (number of times). Moreover, this analysis was calculated using Cohen's *d*, where the mean post-value was subtracted from the mean pre-value and divided by the standard deviation. This method allowed to determine the magnitude of differences obtained with the experimental treatment. The magnitude of the effect was classified as small (*d* = 0.2), intermediate (*d* = 0.5), or large (*d* = 0.8) (Cohen, 1988).

## Results

### Characteristics of the Interventional Studies

**Figure 1:**
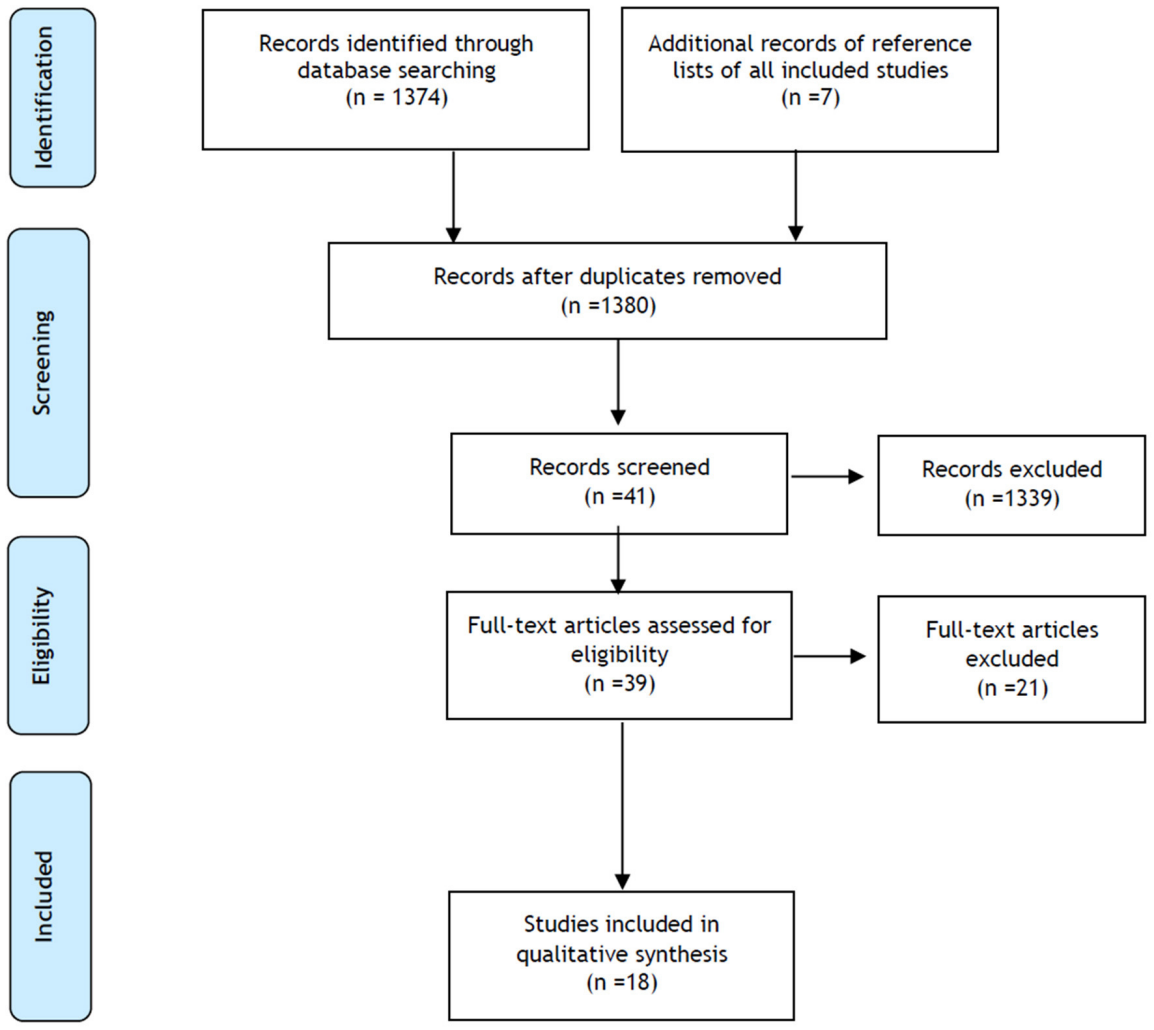
PRISMA (preferred reporting items for systematic reviews and meta-analyses) study flow diagram.

A total of 1,374 records were identified through database searching and 7 additional records were identified through other sources. After removal of duplicates, 1,380 articles were considered for abstract reading. Of the 41 selected articles, only 39 were available as full-text and assessed for eligibility. After full-text reading, 18 studies were included for quality synthesis (Figure 1). Most of the included studies showed intermediate to good quality (Tables S1, S2).

The eighteen (18) included studies collected data from 255 healthy subjects. These participants performed different kinds of exercise interventions (running, cycling, resistance training, and kayaking) in high and/or moderate exercise intensities. The age of the individuals ranged from 18 to 53 years (35.9 ± 17.0). Some of the results were from studies of mixed gender (Abbasi et al., 2013; Stelzer et al., 2015) and the remainder from male volunteers only (Brenner et al., 1999; Mucci et al., 1999; Ostrowski et al., 1999; Connolly et al., 2004; Degerstrøm and Østerud, 2006; Fatouros et al., 2006; Spiropoulos et al., 2010; Bernecker et al., 2011; Nieman et al., 2012; Bonsignore et al., 2013; Draganidis et al., 2013; Marklund et al., 2013; Azizbeigi et al., 2015; Gonzalo-Calvo et al., 2015; Ulven et al., 2015; Wadley et al., 2015). In the studies where the volunteers did more than one bout of exercise in different intensities, the resting period was highly variable: 1 month (Gonzalo-Calvo et al., 2015), 3 weeks (Draganidis et al., 2013), 2 weeks (Brenner et al., 1999), 1 week (Mucci et al., 1999; Ulven et al., 2015; Wadley et al., 2015), or 5 days (Fatouros et al., 2006). The characteristics of the included studies are summarized in Table 2.

**Table 2:** Studies characteristics.

**References**	**Study type**	**Intensity and description of exercise**	**Subjects**	**Inflammatory markers**	**Measure points**	**Findings**
Gonzalo-Calvo et al. (2015)	Cross-sectional	Intense: 10 km-race (89.12% VO_2max_)	9 M Amateur runners Training experience: 6.6 ± 5.0 yr and 69.7 ± 5.0 km/wk	PBMC: WBC, lymphocytes Cytokines: IL-6, IL-8, and IL-10	1.5 h before exer, 10 min, 1 day and 3 days after	↑WBC, Lymphocytes and NK cells 10 min after exer ↑CK from 10 min to 1 day after exer ↔IL-8, IL-6, IL-10, and CRP
		Intense: HM (81.50% VO_2max_)	CRP CK			↑WBC, NK cells, IL-6, IL-10 and CRP 10 min after exer ↑NK cells 10 min and 1 day after exer ↑CK and CRP from 10 min to 1 day and ↓after that ↔Lymphocytes and IL-8
		Intense: Marathon (68.70% VO_2max_)				↑WBC, NK cells, IL-6, IL-8, IL-10 and CRP 10 min after exer ↑CK and CRP from 10 min to 1 day and ↓after that ↔Lymphocytes
Wadley et al. (2015)	Cross-sectional	Intense: LV-HIIE (90% VO_2max_) and high (80% VO_2max_) 10 × 1 min cycling of LV-HIIE with 1 min interval 20 min cycling of high exercise	10 M Untrained	PBMC: Lymphocytes Cytokines: IL-6 and IL-10	Before, at the end and 30 min after exer	↑Lymphocytes at the end, returns to baseline in 30 min ↑IL-10 30 min at the end ↑IL-6 at the end and 30 min after exer
		Moderate: cycled for 27 min(60% VO_2max_)				↑Lymphocytes at the end and returns to baseline in 30 min ↑IL-6 at the end and 30 min after exer ↔IL-10
Ulven et al. (2015)	Cross-sectional	Intense: Cycled for 1 h repeated twice (70% VO_2max_, % HR_max_ ≅ 87.8%, and Borg scale ≅ 15.4)	10 M Very good physical fitness	Cytokines: IL-6, IL-10, and TNF-α	Before and at the end of cycle test	↑IL-6, IL-10, and TNF-α at the end of exer
Azizbeigi et al. (2015)	Controlled Trial	Intense: Resistance training (85–90% of 1 RM) 3 sets of 10–12 repetitions and 1–2 min rest between sets Moderate: Resistance training (65–70% of 1 RM) 3 sets of 3–6 repetitions and 3–4 min rest between sets	30 M (10 control, 10 moderate intensity and 10 high intensity) Untrained but physically active (running, volleyball or soccer)	Cytokines: IL-6 and TNF-α CK	Before, at the end and 3 days after training program	↔IL-6, TNF-α, and CK
Stelzer et al. (2015)	Cross-sectional	Intense: Cycling race (98.68% HR_max_) 8 h of competition and 8 h of rest during 4 days	7 (3 F: 4 M) Moderately trained amateur athletes Training experience 7.5 ± 3.9 h/wk	PBMC: WBC and lymphocytes Cytokines: IL-6 CK	2 days pre-race and 15 min post-race	↑WBC, lymphocytes, IL-6, and CK post-race
Abbasi et al. (2013)	Cross-sectional	Intense: HM in competition conditions (V ≅ 13.26 km/h for men and 11.11 km/h for women) Timing: 95.5 ± 8 min for men and 114 ± 12 min for women	16 (8 F: 8 M) Well-trained athletes Training experience: endurance training for at least 2 yr;	PBMC: WBC and lymphocytes;	Before, 30 min, 3 h and 24 h after exer;	↑WBC at 30 min and 3 h after exer↓Lymphocytes at 30 min and 3 h after exer
Draganidis et al. (2013)	Cross-sectional	Intense: Resistance training: squat, seated leg extension, horizontal leg curls, barbell side lunges, and calf raises; (85–90% 1 RM) 4 sets, 4–6 repetitions per set with 3 min rest Training during around 40–45 min following of 10 min warm-up	10 M Elite football players Training experience: 6 training sessions/wk	PBMC: WBC CRP CK	After, at the end, and daily for 3 days after the exer	↑CRP at the end to 1 day and after returns to baseline ↑CK at 2 days and after returns to baseline ↔WBC
		Moderate: Resistance training: squat, seated leg extension, horizontal leg curls, barbell side lunges, and calf raises (65–70% 1 RM) 4 sets, 8–10 repetitions per set with 1 min rest Training during around 40–45 min following of 10 min warm-up				↑WBC and CRP at 1 day and after returns to baseline Higher elevation in CRP, at the end and 24 h after exer ↑CK at 1 day and after returns to baseline
Marklund et al. (2013)	Cross-sectional	Moderate: 24 h ultra-endurance exer: running, cycling, and kayaking (46–63% VO_2max_); 12 sets of 110 min of exer with 10 min rest for food intake	9 M Well-trained ultra-endurance athletes Training experience: competed in races with long distance (>48 h)	PBMC: WBC Cytokines: IL-6, IL-8, IL-1β, and TNF-α CRP CK	Before, at the end and 28 h after the exer	↑WBC, IL-6 and CK at the end and ↓28 h after exerc ↑IL-8 and CRP at the end and further ↑at 28 h ↔TNF-α and IL-1β
Nieman et al. (2012)	Cross-sectional	Intense: 1.75 h cycling followed by 10 km time trial as fast as possible (18.3 ± 1.7 min) total of 2.1 h cycling (Borg scale = 13.3 ± 1.1 and 82.2 ± 6.1% HR max)	31 M Trained cyclists Training experience: cycling 75 km	PBMC: WBC and lymphocytes Cytokines: TNF-a, IL-6, IL-8, IL-10, and IL-1β	Before, at the end and 1 h post-exer	↑WBC and lymphocyte at the end and ↓1 h after exer ↑TNF- α, IL-6, IL-8, IL-10, and IL-1β at the end of exer ↓TNF-α, IL-6, and IL-8 1 h after; IL-10 and IL-1β continued to ↑1 h after
Bernecker et al. (2011)	Cross-sectional	Intense: Marathon (89.3% HR_max_)	12 M Training experience: Finished HM before	PBMC: WBC Cytokines: IL-6 and TNF- α	Before and 1 h after exer	↑WBC, IL-6, and TNF-α 1 h after exer
Spiropoulos et al. (2010)	Cross-sectional	Intense: Ultra-endurance foot race over a distance of 246 km (9.08 MET by middle age) Finished the race in <36 h	10 M Training experience: done an equal race before	PBMC: WBC Cytokines: IL-6; CRP	Before, at the end and 2 days after race	↑WBC, IL-6 and CRP at the end of exer, IL-6 and WBC return to baseline 2 days after but CRP still ↑
Fatouros et al. (2006)^*^	Cross-sectional	Intense: 4 × 3 wk resistance training period divided in t1, t2, t3, and t4 t1 and t4: low-volume 70 %1 RM (t1 and t4), 75–85% 1 RM (t2) and 85–100% 1 RM (t3); 2 times per wk, 2 sets per exer, 10–12 repeats per set t2: high-volume training (4 times per wk, 4 sets per exer, 6–10 repeats per set) t3: very-high-volume training (6 times per wk, 6 sets per exer, 1–6 repeats per set);	17 M Amateur athletes	CRP CK	Before and 96 h after each session of exer	↑CRP after t2 and t3 ↑CK after t3 and t4
Degerstrøm and Østerud (2006)	Cross-sectional	Intense: 2 sets of 30 min run with 4 h rest (80% VO_2max_)	7 M 5 elite skiers and 3 competing at the district level Training experience: 1 to 2 times/day	PBMC: WBC and lymphocytes Cytokines: IL-6 and IL-8	Before, at the end each run and 2 h after second run	WBC: ↑at the end 1st race; ↓before 2nd race; ↑more at the end 2nd race than 1st; ↓2 h after 2nd race; Lymphocytes and IL-8 ↑after 1st and 2nd run, and IL-8 ↓2 h after 2nd race ↔IL-6
Connolly et al. (2004)	Controlled Trial	Intense: 30 min cycling (80% VO_2max_)	15 M Amateur athletes	PBMC: WBC and lymphocytes Cytokines: IL-6	Before, at the end and 1 h after exer	↑WBC and lymphocytes at the end of exer and return to baseline ↑IL-6 1 h after exer
Bonsignore et al. (2001)	Cross-sectional	Intense: Marathon (15.07 MET by middle age)	8 M Amateur athletes Training experience: 77 ± 15 km/wk and 14 ± 10 yr	PBMC: WBC and lymphocytes	After and at the end of exer	↑WBC at the end of exer↓Lymphocytes at the end of exer
Mucci et al. (1999)	Controlled Trial	Intense: (75and 100% of VO_2max_); Moderate: (50% of VO_2max_); Cycling at 30 Watts for untrained and 60 watts for trained athletes with successive increases of 30 watts every minute (at the end of test the increase was smaller)	22 M 11 highly trained endurance athletes (athletes group) Training experience: 5.2 ± 06 yr and 16.3 ± 1 h/wk	Cytokines: IL-1β and IL-8	Before the exer, at 50% VO_2max_, 75% VO_2max_, 100% VO_2max_ and recovery (5 min after)	↑IL-1β both intensities and returns to baseline in recovery↑IL-8 both intensities and returns to baseline in recovery
			11 untrained group but physically active (control)			↑IL-8 at 100% VO_2max_↔IL-1β
Brenner et al. (1999)^#^	Randomize controlled Trial	Intense: Cycling for 5 min (90% VO_2*max*_)	8 M Moderately fit	PBMC: NK cells and NK Cytolytic activity Cytokines: IL-6, TNF-α and IL-10 CK	30 min before, at the end, 3 h, 1 day and 3 days after each test	↑NK cells at the end of exer and return to baseline 3 h after ↑Citolytic activity at the end of exer; ↓IL-10 from 3 h to 3 days after ↔IL-6, TNF- α, and CK
		Upper limit of moderate: 2 h cycling (60–65% VO_2max_)				↑NK cells at the end of exer and return to baseline 3 h after ↑Citolytic activity at the end of exer ↑IL-6 at the end to 3 h after exer ↑TNF-α from 3 h to 3 days after exer ↔IL-10
		Moderate: 3 sets of 10 repeats in resistance training (bicep curl, knee extension, hamstring, curl, bench press, and leg press) (60–70% 1 RM)				↑NK cells at the end of exer and return to baseline 3 h after ↑CK 3 days after exer ↔IL-10, IL-6, IL-10 and NK cells cytolytic activity
Ostrowski et al. (1999)	Cross-sectional	Intense: Marathon (75.33% VO_2max_)	10 M	Cytokines: TNF-α, IL-1β, IL-6 and IL-10	1 wk before, at the end and every 30 min in the 4 h after exer	↑TNF-α, IL-1β, IL-6 and IL-10 at the end ↓IL-10 and IL-6 (immediately) ↓TNF-α and IL-1β (slowly)

↑, significant increase; ↓, significant decrease; ↔, no change; 1 RM, one repeat maximum; 1st, first; 2nd, second; CK, creatinine kinase; CRP, C-reactive protein; Exer, exercise; F, Female; h, hour; HIGH, high intensity; HM, half marathon; HR_max_, maximum heart rate; IL, interleukin; LV-HIIE, high intensity interval exercise; M, male; PBMC, peripheral blood mononuclear cel; TNF-α, Tumor necrosis factor alpha; V, velocity; VO_2max_, maximum rate of oxygen consumption; WBC, white blood cells; wk, week; yr, years;

* <75% in STROBE quality scale;

# *<75% in CONSORT quality scale*.

The comparison in terms of inflammatory biomarkers between moderate and intense exercise is presented in Table 3. For most studies, blood samples were taken minutes after exercise (immediately, 10 or 15 min). However, some markers were evaluated at other time points: IL- 10 (Wadley et al., 2015), WBC, and lymphocytes (Abbasi et al., 2013) were evaluated also 30 min after exercise; WBC, IL-6, and TNF-α (Bernecker et al., 2011) were evaluated also 1 h after exercise; IL-10 and TNF-α (Brenner et al., 1999) were evaluated also 3 h after exercise and CK (Brenner et al., 1999) was evaluated also 3 days after exercise; CRP and CK (Fatouros et al., 2006) were evaluated also 4 days after exercise. Table 3 considers measurements collected up to 15 min after exercise. The results of IL-6 and CRP from Spiropoulos et al. (2010) were not considered because they showed increases of 10,470 and 6,000 times, respectively, which makes them non-comparable with the other studies. The same occurs with the study by Marklund et al. (2013) in which the baseline value of CRP wasn't detectable.

**Table 3:** Immediate effects of moderate and intense exercise (0–15 min) on inflammatory markers.

**Inflammatory marker**	**Moderate exercise**	**References**	**Intense exercise**	**References**
WBC	↔	Draganidis et al., 2013; Marklund et al., 2013	↑] 1.44; 3.5 [x d = 9.28 ± 6.15^§^	Bonsignore et al., 2001; Connolly et al., 2004; Degerstrøm and Østerud, 2006; Spiropoulos et al., 2010; Nieman et al., 2012; Gonzalo-Calvo et al., 2015; Stelzer et al., 2015
			↔	Bernecker et al., 2011; Abbasi et al., 2013; Draganidis et al., 2013
Lymphocytes	↑1.41 × d = 1.98	Wadley et al., 2015	↑] 1.30; 2.69 [x d = 6.52 ± 4.26	Connolly et al., 2004; Degerstrøm and Østerud, 2006; Nieman et al., 2012; Gonzalo-Calvo et al., 2015; Stelzer et al., 2015; Wadley et al., 2015
			↓4.21 x d = 3.89	Bonsignore et al., 2001
			↔	Abbasi et al., 2013
NK cells	↑5.5 x d = 8.20	Brenner et al., 1999	↑8.83 x d = 10.12	Brenner et al., 1999
NK cells cytolytic activity	↑2.92 x d = 10.48	Brenner et al., 1999	↑4.63 x d = 13.86	Brenner et al., 1999
IL-6	↑] 1.33; 4.20 [x d = 3.91 ± 3.87	Brenner et al., 1999; Wadley et al., 2015	↑] 1.59; 26.79 [x d = 4.04 ± 2.34^§^	Ostrowski et al., 1999; Connolly et al., 2004; Spiropoulos et al., 2010^*^; Nieman et al., 2012;Gonzalo-Calvo et al., 2015; Stelzer et al., 2015; Ulven et al., 2015; Wadley et al., 2015
	↔	Marklund et al., 2013; Azizbeigi et al., 2015	↔	Brenner et al., 1999; Degerstrøm and Østerud, 2006; Bernecker et al., 2011; Azizbeigi et al., 2015
IL-10	↔	Brenner et al., 1999; Wadley et al., 2015	↑] 1.57; 32.99 [x d = 4.55 ± 3.37	Ostrowski et al., 1999; Nieman et al., 2012; Gonzalo-Calvo et al., 2015; Ulven et al., 2015
			↔	Brenner et al., 1999; Wadley et al., 2015
IL-8	↑1.43 x d = 1.04	Mucci et al., 1999	↑] 1.37; 2.77 [x d = 3.63 ± 2.41	Mucci et al., 1999; Degerstrøm and Østerud, 2006; Nieman et al., 2012; Gonzalo-Calvo et al., 2015
	↔	Marklund et al., 2013		
IL-1β	↑1.13 x d = 0.64	Mucci et al., 1999	↑] 1.13; 1.50 [x d = 0.58 ± 0.29	Mucci et al., 1999; Ostrowski et al., 1999; Nieman et al., 2012
	↔	Marklund et al., 2013		
TNF-α	↔	Brenner et al., 1999; Marklund et al., 2013; Azizbeigi et al., 2015	↔	Brenner et al., 1999; Bernecker et al., 2011; Azizbeigi et al., 2015
			↑] 1,30; 2.07 [x d = 2.96 ± 2.20	Ostrowski et al., 1999; Nieman et al., 2012; Ulven et al., 2015
CRP	↑1.23 x d = 1.02	Draganidis et al., 2013; Marklund et al., 2013^#^	↑1.4 x d = 1.15	Spiropoulos et al., 2010^*^; Draganidis et al., 2013
			↔	Fatouros et al., 2006; Gonzalo-Calvo et al., 2015
CK	↑] 1.92; 24.16 [x d = 3.09 ± 1.19	Draganidis et al., 2013; Marklund et al., 2013	↑] 2.19; 4.75 [x d = 3.27 ± 1.89	Draganidis et al., 2013; Gonzalo-Calvo et al., 2015; Stelzer et al., 2015
	↔	Brenner et al., 1999; Azizbeigi et al., 2015	↔	Brenner et al., 1999; Fatouros et al., 2006; Azizbeigi et al., 2015

↑, significant increase; ↓, significant decrease; ↔, no change; CRP, C-reactive protein; d, effect size, expressed as mean ± sd; IL, Interleukin; TNF-α, tumor necrosis factor alpha; WBC, white blood cells;

* Spiropoulos et al. was not included to calculate the increase interval of IL-6 and CRP due very discrepant values after exercise when compared to the other studies (1,0470 and 6,000 times, respectively);

# *Marklund et al. was not included to calculate the increase interval of CRP due baseline value was not detectable*.

§ *Gonzalo-Calvo et al. (2015) and Ostrowski et al. (1999). were not included to calculate d due very discrepant value of 53.03 and 53.18, respectively*.

### Effects of Exercise on Cytokine Secretion

Fifteen studies evaluated the effects of exercise on cytokine concentration in blood (IL-6, IL-8, IL-1β, IL-10, and TNF-α). Our review supports, in general, the idea that exercise can stimulate both pro- and anti-inflammatory responses. These increases were transitory, with the values returning to baseline sometime from 5 to 24 h after exercise.

IL-6 was the cytokine more often evaluated (13 studies) corresponding to 4 moderate and 12 intense exercise bouts. There were increases in IL-6 after exercise ranging from 1.33 to 4.20 times in moderate and from 1.59 to 26.79 in high-intensity exercises, immediately after exercise. In 6 studies there was no increase (2 moderate and 4 intense exercises) (Brenner et al., 1999; Degerstrøm and Østerud, 2006; Bernecker et al., 2011; Azizbeigi et al., 2015).

IL-8 increased after moderate exercise and after intense exercise ranging from 1.37 to 2.77 times (Mucci et al., 1999; Degerstrøm and Østerud, 2006; Nieman et al., 2012; Marklund et al., 2013; Gonzalo-Calvo et al., 2015). IL-10 increased after intense exercise ranging from 1.57 to 32.99 times in 4 studies (Ostrowski et al., 1999; Nieman et al., 2012; Gonzalo-Calvo et al., 2015; Ulven et al., 2015). In three studies there was no increase, one of these referred to intense and moderate exercise, one to moderate and another to intense exercise only.

Gonzalo-Calvo et al. (2015) evaluated the effect of moderate and intense exercise on circulating IL-8, IL-6, and IL-10, and observed an increase in all the evaluated cytokines. This rise was maintained for more than 1 day and then returned to baseline. Wadley et al. (2015) evaluated the cytokine profile after moderate and intense cycling exercise. Their results show an increase in IL-6 at 30 min after exercise independently of intensity. In the same study, IL-10 increases 15 min after intense exercise, without alteration in moderate exercise (Wadley et al., 2015). Mucci et al. (1999) evaluated cycling intense and moderate exercises showing a transient increase in IL-8 in both intensities with a return to baseline values after 5 min. Brenner et al. (1999) evaluated cytokine levels after moderate and intense exercise but only show an increase in IL-6 after moderate exercise (recovery).

Some studies that evaluated only intense exercise showed an increase in IL-6, IL-8, and IL-10; however, values peaked at different times: immediately (15 min) for IL-6 and IL-10 in Ostrowski et al. (1999), 1 or 2 h after exercise for IL-6, IL-8, and IL-10 in Nieman et al. (2012), and for IL-8 in Degerstrøm and Østerud (2006). Marklund et al. (2013) that evaluated moderate ultra-endurance exercise showed an increase in IL-8 and IL-6, 30 min after exercise, however only for IL-8 did this increase remained at 28 h. Spiropoulos et al. (2010) evaluated an intense ultra-endurance exercise and observed an increase of IL-6 maintained at 2 days. The same was observed for the studies by Gonzalo-Calvo et al. (2015), Ostrowski et al. (1999), and Bernecker et al. (2011). These discrepancies might be explained by the duration of the exercises in those studies: the Spiropoulos study refers to ultra-endurance exercise, and the remaining three studies to marathon races (Ostrowski et al., 1999; Bernecker et al., 2011; Gonzalo-Calvo et al., 2015).

Globally the increase in IL-6 and IL-8 levels was higher in intense when compared to moderate exercises. In contrast, IL-10 only showed increases after intense exercise, with no changes after moderate exercise (Figures S1, S2). Nevertheless, the impact of the duration of the exercise bout should be considered when comparing the studies' results.

IL-1β was evaluated in 4 studies with discrepant results (Mucci et al., 1999; Ostrowski et al., 1999; Nieman et al., 2012; Marklund et al., 2013). Mucci et al. (1999) reported approximately the same increase and return to baseline in both exercise intensities for IL-1β. The same pattern was reported in intense exercise by Nieman et al. (2012) and Ostrowski et al. (1999). In contrast, Marklund et al. (2013) reported no changes in this cytokine.

TNF-α was evaluated in 8 studies corresponding to three moderate exercise types without alteration (Brenner et al., 1999; Marklund et al., 2013; Azizbeigi et al., 2015) and six intense exercise types: three with no alteration on the cytokine levels (Brenner et al., 1999; Bernecker et al., 2011; Azizbeigi et al., 2015) and three with increases immediately after exercise (Ostrowski et al., 1999; Nieman et al., 2012; Ulven et al., 2015). All the studies reporting alterations had exercise times of more than 1 h. Only the study by Ostrowski et al. (1999) had repetitive measurements showing a slow decrease in TNF-α values without returning to base levels after 4 h.

### Effects of Exercise on Peripheral Blood Leukocytes

The number of WBC increased after intense exercise in 7 studies (Bonsignore et al., 2001; Connolly et al., 2004; Degerstrøm and Østerud, 2006; Spiropoulos et al., 2010; Nieman et al., 2012; Gonzalo-Calvo et al., 2015; Stelzer et al., 2015). Abbasi et al. (2013) evaluated the effect of high-intensity exercise and observed an increase in WBC numbers 30 min after exercise. This elevation was maintained even after a 3 h recovery period and reflected a pronounced granulocytosis. In contrast, total lymphocyte count had no alterations in the same period but increased 30 min after exercise. All leukocyte counts returned to normal 24 h post-exercise (Abbasi et al., 2013). Similar to this study, Bonsignore et al. (2001) showed an increase of WBC and a decrease of lymphocytes. This increase in WBC occurred due to neutrophils and macrophages.

Nieman et al. (2012) that analyzed the influence of prolonged cycling in high intensity in acute inflammatory response showed an increase in WBC, but, in contrast to previous studies, this occurred due to increased lymphocytes, monocytes, and granulocytes. Lymphocytes and WBC returned to baseline 1 h after exercise. The same pattern occurred in other 3 studies: Degerstrøm and Østerud (2006) with increases at 2 h after exercise, Connolly et al. (2004) with increases at 1 h after exercise and Stelzer et al. (2015) with increases immediately after exercise. These increases in WBC were reportedly due to lymphocytes and granulocytes in the Degerstrøm and Østerud (2006) study; due to neutrophils, monocytes and lymphocytes in the Stelzer et al. (2015) study; and due to lymphocytes and monocytes in the Connolly et al. (2004) study. Other 2 studies of intense exercise also evaluate WBC showing increases after exercise but do not have data on relevant subpopulations (Spiropoulos et al., 2010; Bernecker et al., 2011).

Draganidis et al. (2013) analyzed WBC after resistance training (intense and moderate exercise) showing an increase after moderate exercise, which returns to baseline 1 day after and did not report alterations in WBC after intense exercise. Wadley et al. (2015) evaluated lymphocytes but not total WBC after cycling exercise (moderate and intense) and reported an increase in both intensities that returns to baseline 30 min after exercise. Marklund et al. (2013) evaluated WBC 30 min after moderate exercise showing an increase with a return to baseline values 28 h after exercise.

When comparing both exercise intensities, the increase in total leukocytes only occurs after intense exercise. However, in the lymphocytes subpopulation, both intensities showed similar increases, with only one study presenting a decrease in lymphocytes after intense exercise (Bonsignore et al., 2001).

One study specifically considered NK cell numbers and NK cytolytic activity: Brenner et al. (1999) showed an increase in both parameters after exercise. This increase was greater in intense exercise when compared to moderate exercise (Brenner et al., 1999).

### Effect of Exercise on CK

CK was evaluated in 7 studies corresponding to 10 different exercise types. In short, CK increased in 4 intense type exercises (Fatouros et al., 2006; Draganidis et al., 2013; Gonzalo-Calvo et al., 2015; Stelzer et al., 2015) and 2 moderate exercises (Draganidis et al., 2013; Marklund et al., 2013), with no alteration in the remainder studies evaluating moderate and intense exercise in the same volunteer group. In the study of Draganidis et al. (2013) the increase in CK peaked at 24 h after exercise in the moderate-intensity group, while it peaked at 48 h in the high-intensity group. However, in the study by de Gonzalo-Calvo et al. (2015), CK peaked 24 h after intense exercise and in the study by Marklund et al. (2013) 28 h after moderate exercise. Fatouros et al. (2006) evaluated CRP 4 days after exercise showing an increase. Comparing intense and moderate exercise, the increase in CK was greater in moderate exercise, but only 2 studies evaluate this intensity when compared to 4 studies evaluating the intense exercise.

### Effect of Exercise on CRP

Increases in CRP were observed in one study of moderate exercise (Draganidis et al., 2013) and two studies of intense exercise (Fatouros et al., 2006; Draganidis et al., 2013). In the Draganidis et al. (2013) study the elevation was maintained for 1–2 days before returning to baseline. The increase in CRP on Fatouros et al. (2006) only shows results immediately after exercise. Marklund et al. (2013) evaluating a moderate exercise type, showed an elevation of CRP 30 min after exercise with greater increases 28 h after, but the ratio of the increase cannot be calculated due to the lack of the baseline value. Gonzalo-Calvo et al. (2015) reported elevations of CRP at 24 h after exercise, without alterations before that. Globally, all the studies had increases of this inflammatory marker, with greater values at 24 h. Comparing both exercise intensities, the increase was greater after intense exercise, but only one study referred to moderate-intensity exercise.

## Discussion

This systematic review evaluated the changes of inflammatory markers after moderate and intense exercise bouts. The findings of the current review suggest that there is an acute inflammation profile after exercise, with the increase of most inflammatory markers, especially in high-intensity exercise. In samples taken immediately after long and intense exercises we could not rule out the effects of dehydration on the plasma volume, and hence the quantification of the inflammatory markers measured.

### Exercise-Induced Effects on Cytokines

Results suggested that there are substantial discrepancies in the extent of pro-inflammatory changes in the immune system. By examining the impact of different intensity exercise on anti-inflammatory cytokines secretion, some of the studies showed an increase while others report no change (Brenner et al., 1999; Bernecker et al., 2011; Marklund et al., 2013; Azizbeigi et al., 2015). This might be a consequence of cytokines appearing only transiently in the blood and thereby evading detection. Moreover, cytokines are secreted by many cells and tissues, with muscle considered to be a major contributor during exercise, as such, circulating levels might not reflect levels in source tissues.

IL-6 concentration increases more than other cytokines during exercise which might indicate muscle damage (Allen et al., 2015; Lightfoot and Cooper, 2016). IL-6 plasma concentrations are reportedly affected by factors other than intensity, such as type and time of exercise (Gleeson et al., 2011; Baumert et al., 2016). Persistent elevation of this cytokine can be associated with muscle atrophy, that results in a reduction on strength and muscle function and increased muscle pain (Hennigar et al., 2017). Following acute exercise, elevated levels of IL-6 promote an increase in IL-10 and IL-1RA, two anti-inflammatory cytokines.

Most studies showed an increase in IL-10 after intense exercise which is consistent with previous reports (Moldoveanu et al., 2001; Allen et al., 2015). Shaw et al. (2017) demonstrated that IL-10 production, after strenuous acute exercise, is equivocal, with increases, decreases and no changes in exercises with different protocols and analytical techniques. Regardless, levels of IL-10 tend to peak during recovery time from exercise, with the magnitude of the increase being related to the active muscle mass and exercise intensity. Overall, the duration of the exercise was the most important factor determining the magnitude of the exercise-induced increase of plasma IL-10, as recently reviewed by Santos et al. (2019). This increase in IL-10 could be related to the prevention of potential deleterious chronic low-grade inflammation and tissue damage.

TNF-α was only stimulated by intense endurance exercise (more than 1 h) (Ostrowski et al., 1999; Nieman et al., 2012; Ulven et al., 2015). In contrast to our main findings, Starkie et al. (2003) showed that the production of TNF-α decreased after a single endurance exercise bout and Moldoveanu et al. (2001) reported an increase in TNF-α after three 2–3 h moderate bouts (cycling and run + cycling). However, no change was found after a single 45 min moderate bout or one 5 min intense bout (Moldoveanu et al., 2001). These results suggest that other factors, namely exercise duration, are also important in regulating TNF-α release.

Previous reports suggested that IL-1 release depends on the type, intensity and duration of exercise (Moldoveanu et al., 2001). The current review shows that IL-1β increases in all studies of intense exercise but in none after moderate exercise. Similar to TNF-α, the local increase of IL-1 is higher than the systemic increase, after eccentric exercise (Baumert et al., 2016). IL-1β is a potent pro-inflammatory cytokine, influencing adhesion molecules and chemokines, and by this, possibly relating to leukocyte migration and function.

In this systematic review, it was observed that IL-8 increased after both exercise intensities, which is consistent with previous reviews that report increased IL-8 systemic levels associated with damaging exercise regimes (Moldoveanu et al., 2001; Lightfoot and Cooper, 2016). Suzuki et al. (2003) showed that IL-8 increases after prolonged exercise, with some alteration after acute intense exercise. The same occurs in this systematic review, with IL-8 systemic levels increasing after prolonged and acute intense exercises.

In general, cytokines increase more with intense than with moderate exercise, but these increases are not consistent, being influenced by the duration and type of exercise.

### Exercise-Induced Effects on WBC

Peake et al. (2017) showed that a single bout can cause changes in blood number of leukocytes, which persist during exercise recovery and in contrast, decrease faster (in 30 min) after especially prolonged and/or intense exercise. The included studies showed a WBC increase immediately after intense exercise; however, in the recovery period, the decrease was inconsistent between studies. WBC number was the only inflammatory marker studied that showed a clear increase after intense exercise in all the included studies, without alteration in moderate exercise studies. The results also suggest a chronology in the mobilization of the different leukocyte populations to the blood, with lymphocytosis occurring at the end of the intense exercise bouts and decreasing shortly after (30 min) (Wadley et al., 2015).

As a part of the innate immune system, NK cells can recognize and eliminate neoplastic and virus-infected cells without prior contact (Bigley and Simpson, 2015). The decrease in NK activity is accompanied by an increased incidence of infectious diseases (Fu et al., 2014). NK cell number and NK cell activity increased after moderate and intense exercise, but this was verified in only one study included in this systematic review. The study by Brenner et al. (1999) showed that acute physical exercise increased the activity and induced mobilization of NK cells to the peripheral blood independently of exercise intensity. Theoretically, a high frequency of NK cells can possibly protect the body against infections or tumor progression, but it must be bared in mind that these increases were transitory, and possible migration of NK cells to peripheral tissues was not assessed. Previous reviews reported NK and NK activity increases in response to stressors (Timmons and Cieslak, 2008; Viana et al., 2014; Bigley and Simpson, 2015; Peake et al., 2017).

Studies that explore the true effect of changes in cell distribution in response to exercise and health status are lacking, and the relevance of these findings cannot be fully appraised as we failed to consider the number of cells infiltrating in the muscle tissue in response to exercise. In fact, Marklund et al. (2013) showed that a moderate-intensity endurance activity (~60% V02 peak) sustained during a very prolonged period induced an extensive local muscle infiltration.

### Exercise-Induced Effects on CK

CK results were very discrepant, as half of the studies showed no alterations immediately after intense or moderate exercise, while the other half showed an increase of CK levels. Moghadam-Kia et al. (2016) referred that CK levels have a significant variation with sex and race. The degree of this change depended on the duration and type of exercise; with strenuous exercise being responsible for greater elevations. Damaged muscle fiber structures were pointed out as being the cause for the rise, but one study of repeated eccentric exercise caused almost no increase on CK levels (Baumert et al., 2016). CK was the only marker whose increase was higher in moderate when compared to intense exercise, but few studies were available in the moderate exercise arm. In general, the muscle damage, as evidenced by CK activity was not accompanied by parallel increases in inflammatory markers, namely cytokines and CRP.

### Exercise-Induced Effects on CRP

Of the proteins stimulated during the acute phase response, CRP has received the most attention as a marker of inflammation in both rheumatic and non-rheumatic diseases (Petersen and Pedersen, 2005; Schrödl et al., 2016). It is proposed to have a scavenger function to eliminate bacterial products or damaged cells and to attenuate the consequences of infection or tissue injury. Petersen and Pedersen (2005) reported that this inflammatory marker has a role in the suppression of the synthesis of pro-inflammatory cytokines by tissue macrophages and in the induction of anti-inflammatory cytokines. Because the levels of CRP increase dramatically during inflammation processes and remain elevated for a long period of time CRP can be a suitable marker. In this systematic review, two studies (Fatouros et al., 2006; Draganidis et al., 2013) had an increase in CRP at the end of exercise in contrast of Petersen and Pedersen (2005) where CRP increased 1 day after. This discrepancy may be due to the fact that in those studies the athletes did short sets of exercise and in Petersen and Pedersen (2005) they practice longer duration exercises. Fedewa et al. (2016) indicated that CRP had a significant and small decrease following training, but doesn't report values immediately after acute exercise. This information is in opposition to Petersen and Pedersen (2005), showing that regular exercise induces a reduction in CRP.

### Implications for Practice

Exercise has been established as a part of multimodal therapeutic approaches in several pathologies contributing to cardiorespiratory fitness, muscle strength, flexibility, and neuromotor performance. However, the strong variability in study designs, type, duration, and intensity of exercise remain obstacles in the assessment of the measurable effects of exercise on inflammatory markers.

A recent systematic review on the impact of physical activity on serum levels of inflammatory markers in rheumatoid arthritis patients failed to conclude that there is a significant impact on systemic levels of inflammatory markers (Burghardt, 2019). Nevertheless, attention is needed when recommending and prescribing physical activity to these specific patients. One should be aware of the possible influence of medication, and the potential increase of pain and disease activity by performing physical activity, even without any changes of inflammatory markers.

Previous studies suggested that the effect of aerobic exercise on the cytokines' increase is different from the effect of strength/resistance exercise, with the latter being less evident (Santos et al., 2019). This is in line with our evidence. Moreover, strength/resistance exercises are influenced by different variables such as intensity, workload, number of repetitions, the interval between sets, and size of muscle mass involved in muscle contraction. Whereas, the anti-inflammatory nature of IL-6 contributes to the acute phase response and the adaptation of skeletal muscle to exercise, chronically elevated levels of IL-6 contribute to persistent inflammation and muscle wasting (Lightfoot and Cooper, 2016). The release of anti-inflammatory mediators, such as IL-10, as a compensatory mechanism, might also impair immune responses. The pronounced anti-inflammatory response induced by prolonged and exhaustive exercise could lead to transient suppression of several immune components and increase the risk of infection (Shaw et al., 2017).

### Limitations

The results of this review were based on individual sports, such as cycling, resistance training, and running, which limits its application to other types of sports. Some limitations were found in the compilation and comparison of results because the time, type of exercise, and a number of bouts were different among studies. In addition, we did not perform a comparative analysis (meta-analysis), because such analysis could not be easily accomplished due to the lack of consistency in parameters and the lack of uniformity. Because of the non-response of some study's authors, some articles with important findings might not be included. CRP was the most restrictive inflammatory marker, with no possible comparison of concentrations since measurement methods varied widely. Our findings showed that most studies follow the same pattern of changes; however, the amplitude of those changes at the systemic level does not always correlate with exercise-induced changes in local inflammation. Another limitation is that most of the studies performed the experiment at a single level of intensity with a relatively small number of participants, which might have contributed to increasing the individual variability. All the studies included in this systematic review refer to healthy non-sedentary individuals. As such, it is not possible to ascertain if the same results would be valid for sedentary individuals that initiate exercise practice and what would the implications be in populations with chronic inflammatory pathologies. Our conclusions might also have been limited by restricting our search to the PubMed database, as other relevant studies might have not been considered.

## Conclusion

Based on the current review findings, exercise has considerable effects on inflammation markers. Pro-inflammatory cytokine TNF- α and anti-inflammatory IL-10 only increase after intense exercise, and pro-inflammatory cytokines IL-6 and IL- 1β increase more with intense than with moderate exercise. The main differences regarding the effect of intensity of exercise on the inflammation markers studies were found in total WBC, IL-6, and IL-10, with higher increases in intense than in moderate exercise bouts. The highest alterations occur after intense exercise in IL-6 with increases up to 26.79 times and in IL-10 with increases up to 32.99 times, corresponding a VO_2max_ of 75.33% In moderate exercise studies, higher alterations occur in CK with an increase of 24.16 times at a VO_2max_ of 46–63%. However, our results were not consistent, with discrepancies probably due to the emphasis on muscle contraction (eccentric vs. concentric) and intensity of the effort related to the type of the exercise. Nevertheless, and although regular exercise presents a global positive anti-inflammatory effect, high-intensity exercise, especially when performed with reduced recovery periods, induces a persistent dysregulation of the immune system with increased susceptibility to illness. Further research is required to examine the impact of exercise intensity on inflammation. It is important that future studies carefully assess not only intensity but also associate it with exercise type and duration, as those aspects were found to deeply influence inflammation within the intense exercise group.

## Author Contributions

ÉC and OL carried out literature searches and wrote the manuscript's first draft. DM and HN checked the sports aspects and the potential training implications. OL checked the immunological aspects. ÉC, DM, HN, and OL checked the final version of the manuscript. All authors made contributions to the review.

### Conflict of Interest

The authors declare that the research was conducted in the absence of any commercial or financial relationships that could be construed as a potential conflict of interest.

## Footnotes

^1^NCBI Pubmed. Available online at: https://www.ncbi.nlm.nih.gov/pubmed/

## Abbreviations

CK: creatine kinase
CONSORT: Consolidated Standards of Reporting Trials
CRP: C-reactive protein
HR_max_: maximal heart rate
HRR_max_: Maximal heart rate reserve
IL: Interleukin
MEDLINE: MEDical Literature Analysis and Retrieval System
MET: Metabolic equivalent
NK: Natural killer
PBMC: Peripheral Blood Mononuclear Cells
PICO, Population, Intervention: Comparison and Outcome
PRISMA: Preferred Reporting Items for Systematic reviews and Meta-Analysis
RM: Repetition maximum
STROBE: Strengthening the reporting of observational studies in epidemiology
TNF-α: Tumor necrosis factor alpha
VO_2max_: Maximal oxygen consumption
WBC: White Blood Cells.
