# Supplementary material for: Inflammatory Effects of High and Moderate Intensity Exercise—A Systematic Review
Source: Front Physiol. 2020 Jan 9;10:1550. doi: 10.3389/fphys.2019.01550 (PMC6962351; doi:10.3389/fphys.2019.01550)
Supplement: Supplementary file 1 [file Table_1.DOCX]

**Supplementary Tables and figures**

Table S1 – Quality assessment of the cross-sectional studies using the STROBE scale (20)

|  |  | Autor, year | | | | | | | | | | | | | | | |
| --- | --- | --- | --- | --- | --- | --- | --- | --- | --- | --- | --- | --- | --- | --- | --- | --- | --- |
|  |  | Gonzalo-Calvo, 2015 | Wadley, 2015 | Ulven, 2015 | Stelzer, 2014 | Abbasi, 2013 | Draganidis, 2013; | Marklund, 2013; | Nieman, 2012; | Spiropoulos, 2010; | Stewart, 2007; | Fatouros, 2006; | Degerstrom, 2006; | Ostrowski, 1999; | Bernecker, 2011 | Xiang, 2014; | Bonsignore, 2001; |
|  |  |  |  |  |  |  |  |  |  |  |  |  |  |  |  |  |  |
| STROBE scale Itens | 1 (a) | × | × | × | × | × | × | × | × | × | × | × | × | × | × | × | × |
|  | 1 (b) | ✓ | ✓ | ✓ | ✓ | ✓ | ✓ | ✓ | ✓ | ✓ | ✓ | ✓ | ✓ | ✓ | ✓ | ✓ | ✓ |
|  | 2 | ✓ | ✓ | ✓ | ✓ | ✓ | ✓ | ✓ | ✓ | ✓ | ✓ | ✓ | ✓ | ✓ | ✓ | ✓ | ✓ |
|  | 3 | ✓ | ✓ | ✓ | ✓ | ✓ | ✓ | ✓ | ✓ | ✓ | ✓ | × | ✓ | ✓ | × | ✓ | ✓ |
|  | 4 | ✓ | ✓ | ✓ | ✓ | ✓ | ✓ | ✓ | ✓ | ✓ | ✓ | ✓ | ✓ | ✓ | ✓ | ✓ | ✓ |
|  | 5 | ✓ | ✓ | ✓ | ✓ | ✓ | ✓ | ✓ | ✓ | ✓ | ✓ | ✓ | ✓ | ✓ | ✓ | ✓ | ✓ |
|  | 6 (a) | ✓ | ✓ | ✓ | ✓ | ✓ | ✓ | ✓ | ✓ | ✓ | ✓ | ✓ | ✓ | × | × | ✓ | ✓ |
|  | 7 | ✓ | ✓ | ✓ | ✓ | ✓ | ✓ | ✓ | ✓ | ✓ | ✓ | ✓ | ✓ | × | × | ✓ | ✓ |
|  | 8 | ✓ | ✓ | ✓ | ✓ | ✓ | ✓ | ✓ | ✓ | ✓ | ✓ | ✓ | ✓ | ✓ | ✓ | ✓ | ✓ |
|  | 9 | ✓ | ✓ | ✓ | ✓ | ✓ | ✓ | ✓ | ✓ | ✓ | ✓ | × | ✓ | ✓ | ✓ | ✓ | ✓ |
|  | 10 | ✓ | ✓ | ✓ | ✓ | ✓ | ✓ | ✓ | ✓ | ✓ | ✓ | ✓ | ✓ | ✓ | ✓ | ✓ | ✓ |
|  | 11 | ✓ | ✓ | ✓ | ✓ | ✓ | ✓ | ✓ | ✓ | ✓ | ✓ | ✓ | ✓ | ✓ | ✓ | ✓ | ✓ |
|  | 12 (a) | ✓ | ✓ | ✓ | ✓ | ✓ | ✓ | ✓ | ✓ | ✓ | ✓ | ✓ | ✓ | ✓ | ✓ | ✓ | ✓ |
|  | 12 (b) | ✓ | ✓ | ✓ | ✓ | ✓ | ✓ | ✓ | ✓ | ✓ | ✓ | ✓ | ✓ | ✓ | ✓ | ✓ | ✓ |
|  | 12 (c) | — | — | — | — | — | — | — | — | — | — | — | — | — | — | — | — |
|  | 12 (d) | ✓ | ✓ | × | × | × | × | × | × | × | × | × | × | × | × | × | × |
|  | 12 (e) | ✓ | ✓ | ✓ | ✓ | ✓ | ✓ | ✓ | ✓ | ✓ | ✓ | ✓ | ✓ | ✓ | ✓ | ✓ | ✓ |
|  | 13 (a) | ✓ | ✓ | ✓ | ✓ | ✓ | ✓ | ✓ | ✓ | ✓ | ✓ | ✓ | ✓ | ✓ | ✓ | ✓ | ✓ |
|  | 13 (b) | ✓ | — | ✓ | ✓ | — | ✓ | — | — | — | — | — | — | — | ✓ | — | ✓ |
|  | 13 (c) | ✓ | — | × | × | — | × | — | — | — | — | — | — | — | × | — | × |
|  | 14 (a) | ✓ | ✓ | ✓ | ✓ | ✓ | ✓ | ✓ | ✓ | ✓ | ✓ | ✓ | ✓ | ✓ | ✓ | ✓ | ✓ |
|  | 14 (b) | — | — | ✓ | — | — | — | — | — | — | — | — | — | — | — | — | — |
|  | 15 | ✓ | ✓ | ✓ | ✓ | ✓ | ✓ | ✓ | ✓ | ✓ | ✓ | ✓ | ✓ | ✓ | ✓ | ✓ | ✓ |
|  | 16 (a) | ✓ | ✓ | ✓ | ✓ | ✓ | ✓ | ✓ | ✓ | ✓ | ✓ | × | ✓ | ✓ | ✓ | ✓ | ✓ |
|  | 16 (b) | ✓ | ✓ | ✓ | ✓ | ✓ | ✓ | ✓ | ✓ | ✓ | × | × | × | × | ✓ | × | ✓ |
|  | 16 (c) | — | — | — | — | — | — | — | — | — | — | — | — | — | — | — | — |
|  | 17 | ✓ | ✓ | ✓ | ✓ | ✓ | × | ✓ | ✓ | × | ✓ | ✓ | ✓ | ✓ | ✓ | ✓ | ✓ |
|  | 18 | ✓ | ✓ | ✓ | ✓ | ✓ | ✓ | ✓ | ✓ | ✓ | ✓ | ✓ | ✓ | ✓ | ✓ | ✓ | ✓ |
|  | 19 | ✓ | × | ✓ | ✓ | ✓ | ✓ | × | × | × | × | × | × | × | ✓ | ✓ | ✓ |
|  | 20 | ✓ | ✓ | ✓ | ✓ | ✓ | ✓ | ✓ | ✓ | ✓ | ✓ | ✓ | ✓ | ✓ | ✓ | ✓ | ✓ |
|  | 21 | ✓ | ✓ | ✓ | ✓ | ✓ | ✓ | ✓ | ✓ | ✓ | ✓ | ✓ | ✓ | ✓ | ✓ | ✓ | ✓ |
|  | 22 | ✓ | ✓ | ✓ | ✓ | ✓ | ✓ | × | ✓ | ✓ | ✓ | ✓ | × | ✓ | ✓ | × | ✓ |
|  | Total | 96.55% | 92.59% | 90 % | 89.66% | 92.59% | 86.21% | 85.19% | 88.89% | 85.19% | 85.19% | 74.07% | 81.48 % | 77.78 % | 79.31% | 85.19 % | 89.66 % |

✓: yes; ×: no; —: not applicable; studies with a compliance percentage of the STROBE scale above 75% were considered to have good quality, studies with a compliance percentage between 50 and 75% were considered to have an average quality, studies with a compliance percentage below 50% were considered to have a low quality.

Table S2 - Quality assessment of the clinical trials using the CONSORT (19)

|  |  | Autor, year | | | |
| --- | --- | --- | --- | --- | --- |
|  |  | Azizbeigi, 2015 | Mucci, 1999B | Brenner, 1999 | Connolly, 2004 |
| CONSORT scale Itens | 1 (a) | × | × | × | × |
|  | 1 (b) | ✓ | ✓ | ✓ | ✓ |
|  | 2 (a) | ✓ | ✓ | ✓ | ✓ |
|  | 2 (b) | ✓ | ✓ | ✓ | ✓ |
|  | 3 (a) | ✓ | ✓ | ✓ | × |
|  | 3 (b) | ✓ | — | — | — |
|  | 4 (a) | ✓ | ✓ | ✓ | ✓ |
|  | 4 (b) | ✓ | ✓ | ✓ | ✓ |
|  | 5 | ✓ | ✓ | ✓ | ✓ |
|  | 6 (a) | ✓ | ✓ | ✓ | ✓ |
|  | 6 (b) | — | — | — | — |
|  | 7 (a) | ✓ | ✓ | ✓ | ✓ |
|  | 7 (b) | — | — | — | — |
|  | 8(a) | × | × | × | × |
|  | 8(b) | ✓ | × | × | × |
|  | 9 | × | × | × | × |
|  | 10 | × | × | × | × |
|  | 11 (a) | — | — | — | — |
|  | 11 (b) | — | — | — | — |
|  | 12(a) | ✓ | ✓ | ✓ | ✓ |
|  | 12(b) | ✓ | ✓ | ✓ | ✓ |
|  | 13 (a) | — | — | — | — |
|  | 13(b) | — | — | — | — |
|  | 14 (a) | ✓ | ✓ | ✓ | ✓ |
|  | 14(b) | — | — | — | — |
|  | 15 | ✓ | ✓ | × | ✓ |
|  | 16 | ✓ | ✓ | ✓ | ✓ |
|  | 17 (a) | ✓ | ✓ | ✓ | ✓ |
|  | 17 (b) | ✓ | ✓ | × | ✓ |
|  | 18 | ✓ | ✓ | ✓ | — |
|  | 19 | — | — | — | — |
|  | 20 | ✓ | ✓ | ✓ | ✓ |
|  | 21 | ✓ | ✓ | ✓ | ✓ |
|  | 22 | ✓ | ✓ | ✓ | ✓ |
|  | 23 | ✓ | ✓ | ✓ | ✓ |
|  | 24 | ✓ | — | — | ✓ |
|  | 25 | ✓ | ✓ | ✓ | ✓ |
|  | Total | 86.66% | 80.77% | 73.08% | 76.92% |

✓: yes; ×: no; —: not applicable; studies with a compliance percentage of the STROBE scale above 75% were considered to have good quality, studies with a compliance percentage between 50 and 75% were considered to have an average quality, studies with a compliance percentage below 50% were considered to have a low quality.

Figure S1 - IL-10 concentration with time, in different studies. Pre: before exercise; Post: immediately after exercise

Figure S2 - IL-6 concentration with time, in different studies. Pre: before exercise; Post: immediately after exercise
